# Acute Kidney Injury in Pediatric Diabetic Kidney Disease

**DOI:** 10.3389/fped.2021.668033

**Published:** 2021-06-15

**Authors:** Federica Piani, Trenton Reinicke, Claudio Borghi, Kalie L. Tommerdahl, Gabriel Cara-Fuentes, Richard J. Johnson, Petter Bjornstad

**Affiliations:** ^1^Division of Renal Diseases and Hypertension, Department of Medicine, University of Colorado Anschutz Medical Campus, Aurora, CO, United States; ^2^Section of Pediatric Endocrinology, Department of Pediatrics, Children's Hospital Colorado and University of Colorado Anschutz Medical Campus, Aurora, CO, United States; ^3^Department of Medicine and Surgery Sciences, Alma Mater Studiorum–University of Bologna, Bologna, Italy; ^4^Section of Pediatric Nephrology, Department of Pediatrics, Children's Hospital Colorado, Aurora, CO, United States

**Keywords:** acute kidney injury, diabetes, diabetic kidney disease, pediatric, COVID-19

## Abstract

Diabetic kidney disease (DKD) is a common complication of type 1 and 2 diabetes and often presents during adolescence and young adulthood. Given the growing incidence of both type 1 and type 2 diabetes in children and adolescents, DKD represents a significant public health problem. Acute kidney injury (AKI) in youth with diabetes is strongly associated with risk of DKD development. This review will summarize the epidemiology and pathophysiology of AKI in children with diabetes, the relationship between AKI and DKD, and the potential therapeutic interventions. Finally, we will appraise the impact of the recent COVID-19 infection pandemic on AKI in children with diabetes.

## Introduction

Type 1 diabetes (T1D) has consistently remained one of the most common chronic diseases affecting children and adolescents (1). In the past few decades, the incidence of youth-onset type 2 diabetes (T2D) has also progressively increased, likely due to rising rates of childhood obesity (2). Both types of diabetes are associated with long term complications that result in increased morbidity and mortality (1). Among these complications, diabetic kidney disease (DKD) is associated with the highest rates of excess mortality observed in young persons with diabetes (1). Indeed, DKD represents the leading cause of end-stage kidney disease (ESKD) and dialysis in the developed world (3). The pathophysiology of DKD is multifaceted and is characterized by progressive chronic kidney disease (CKD) (1, 4). In children and adolescents with diabetes, AKI can magnify the risk for CKD development and progression later in life (5–9).

Several mechanisms have been proposed to explain the accentuated risk of acute kidney injury (AKI) in youth with diabetes, including diabetic ketoacidosis (DKA), acute hyperglycemic events, and chronic poor glycemic control (10–12). Hyperglycemia has been shown to directly induce kidney inflammation and tubulopathy (12), while poor glycemic control can lead to polyuria with resultant volume contraction and hypovolemia, which is subsequently associated with the development of pre-renal AKI (11). In this review we seek to appraise the evolving mechanisms, risk-factors, and management strategies for diabetes-induced AKI in the pediatric population.

## Epidemiology and Pathophysiology of AKI in Diabetes

### Epidemiology and Definition of AKI in the Pediatric Population

AKI in youth represents a significant and growing challenge for clinicians, as AKI has been demonstrated in 3.9 out of every 1,000 pediatric hospitalizations at-risk in the U.S. (13), including up to 64% of hospitalizations for DKA in youth with T1D (14). AKI is currently defined by the Kidney Disease Improving Global Outcomes (KDIGO) consensus classification based on conventional serum creatinine and urine output (UO) criteria (15). Previous widely used classification criteria have also shown an excellent accuracy in screening for AKI in the pediatric population. The pediatric Risk, Injury, Failure, Loss and End-stage Kidney (pRIFLE) criteria, which include a decrease in estimated creatinine clearance (eCCl) per the Schwartz formula over 8 to 24 h and anuria for 12 h (15), and the Acute Kidney Injury Network (16) criteria, which include an increase in serum creatinine over 6 to 24 h and anuria for 12 h (15). The pRIFLE criteria have been shown to have high sensitivity in detecting AKI (16), and the AKIN criteria have demonstrated high specificity (15).

AKI is typically classified into three main categories: pre-renal, intrinsic/renal, and post-renal (17). The most common form of pediatric AKI is pre-renal, a usually reversible form of kidney dysfunction caused by kidney hypoperfusion (18, 19). In the setting of diabetes, the extracellular volume depletion leading to pre-renal AKI is commonly induced by glycosuria because of poorly controlled diabetes (14, 20). The combination of poor glycemic control with pre-renal AKI can eventually lead to intrinsic renal AKI, characterized by structural damage to the renal parenchyma and the occurrence of tubular necrosis (18, 21). Although DKD has historically been considered a glomerular disease, a growing body of evidence suggests that tubular-interstitial injury may be the first alteration in DKD (18, 21). AKI is also divided by severity into stage 1, 2, or 3 with different definitions according to the applied diagnostic criteria for AKI (e.g., pRIFLE AKI stage 1 is defined as a 25% decrease of estimated GFR (eGFR), stage 2 as a decrease of 50%, and stage 3 as a decrease of 75%) (15). Despite a growing body of literature evaluating the incidence and etiologies of AKI in adults, large epidemiologic studies involving pediatric populations with AKI, with or without diabetes, are lacking, as many studies are either limited to a single center or are focused on specific subpopulations (13, 18) (Table 1).

**Table 1:** Studies on AKI in pediatric populations with and without diabetes.

**Study**	***n***	**Study population**	**Study aim**	**Results**
Askenazi et al. (8)	174	Children who had previously developed AKI at a single hospital	3–5-year survivorship among children hospitalized with AKI	The 3–5-year survivorship after hospitalization among children with an episode of AKI was 139/174 (79.9%). Thus, patients have a high risk of ongoing residual renal injury and death after AKI
Mammen et al. (9)	126	Children who survived an episode of AKI at a tertiary-care pediatric intensive care unit from 2006 to 2008	Determine the incidence of CKD development following an episode of AKI	13/126 (10.3%) of children developed CKD 1–3 years after AKI. In addition, 59/126 (46.8%) patients were identified as being at risk of CKD
Sutherland et al. (13)	2,644,263	Children in the United States (U.S.) listed in the 2009 Kids Inpatient Database	Characterize pediatric AKI across the U.S. and identify AKI risk factors among a national cohort	AKI occurs in 3.9/1,000 at-risk pediatric hospitalizations. Mortality was highest among neonates and children requiring critical care or dialysis. AKI occurs most commonly with systemic/multiorgan disease
Hursh et al. (14)	165	Children with T1D hospitalized for DKA at a single hospital from 2008 to 2013	Determine the proportion of children hospitalized for DKA who develop AKI, as well as the associated markers of AKI	106/165 (64.2%) of children admitted for DKA had developed AKI. AKI was associated with clinical and biochemical markers of volume depletion and severe acidosis
Kaddourah et al. (22)	4,683	Multinational, prospective study involving pediatric patients admitted to pediatric intensive care units	Define the incremental risk of death and complications associated with severe AKI	AKI developed in 1,261/4,683 patients (26.9%) and severe AKI developed in 543/4,683 patients (11.6%). Death occurred in 60/543 patients (11.0%) with severe AKI vs. 105/4,140 patients (2.5%) without severe AKI. AKI is common and associated with poor outcomes, including increased mortality, among critically ill children and young adults
Baalaaji et al. (23)	79	Children with DKA admitted to a single pediatric intensive care unit (PICU) between 2011 and 2014	Identify the predictors and outcomes of AKI in children	28/79 (35.4%) children developed AKI. 20/28 (71.4%) children with AKI recovered with hydration alone. Serum chloride at 24 h was independently associated with AKI. Children with AKI had prolonged acidosis, longer PICU stay, and higher mortality
Ho et al. (24)	74	Children admitted to a single children's hospital with DKA, with and without AKI, from 2010 to 2018	Assess the influence of intravenous fluid regimens and blood pH on the incidence of AKI in pediatric DKA	There was no statistically significant difference between the volume of IV fluid given to patients with AKI and those without AKI
Charlton et al. (25)	2,110	Neonates admitted to a neonatal intensive care unit who received at least 48 h of intravenous fluids	To assess the risk factors and outcomes of neonatal AKI in the first postnatal week	AKI in the first postnatal week is common and associated with death and longer duration of hospitalization. Risk factors for AKI included resuscitation with epinephrine, admission diagnosis of hyperbilirubinemia, inborn errors of metabolism, and surgical need
Myers et al. (26)	1,255	Children admitted to the Emergency Department with a diagnosis of DKA in 13 United States hospitals	Investigate risk factors for AKI and its association with neurocognitive outcomes in pediatric DKA	AKI occurred in 584/1,359 (43.0%) of DKA episodes. Children with AKI, when compared to those without, had lower scores on tests of short-term memory during DKA. AKI may occur more frequently in children with greater acidosis and circulatory volume depletion during DKA
Hapca et al. (27)	16,700	Retrospective, cohort study of participants with or without T2D over a median period of 8.2 years	Evaluate rates of AKI and to determine their relationship to CKD status and further kidney function decline	Patients with diabetes have significantly higher rates of AKI compared to patients without diabetes. In addition, patients with diabetes were significantly more likely to have preexisting CKD or CKD that developed during follow-up
Yang et al. (28)	58	Retrospective study performed in a single center from 2004 to 2018 including children admitted with DKA who had T1D	Assess incidence and clinical characteristics of AKI and to identify the associated risk factors of AKI in children with T1D and DKA	AKI frequently occurred in children with T1D who had DKA. Longer duration of TID and elevated anion gap are associated with occurrence of severe AKI
DePiero et al. (29)	1,389	Children diagnosed with DKA in 13-centers from 2011 to 2016	Characterize hemodynamic alterations occurring during DKA and to identify clinical and biochemical factors associated with hypertension	Hypertension occurs in a substantial number of children with DKA (27.8%). Factors associated with hypertension include severe acidosis, AKI, and lower Glasgow Coma Scale scores
Williams et al. (30)	66	Children with DKA in a tertiary care, teaching, and referral hospital	To investigate 0.9% saline compared to Plasma-Lyte-A as an initial fluid in pediatric DKA	The incidence of new or progressive AKI and resolution of AKI were similar in both groups. Plasma-Lyte-A was similar to 0.9% Saline in time to resolution of DKA, need for renal replacement therapy, mortality, and lengths of pediatric intensive care unit and hospital stay
Huang et al. (10)	223	Children presenting with T1D or T2D and DKA between 2000 and 2017	Identify the prevalence of AKI and associations between AKI severity and recovery time from metabolic acidosis	170/223 (56.5%) patients with DKA presented with AKI. Approximately 80% of children with DKA recovered from metabolic acidosis on the first day, regardless of AKI severity
De Zan et al. (31)	811	Children admitted to the pediatric intensive care unit at a single center from 2014 to 2016	Assess the incidence rate of AKI, identify risk factors, and evaluate clinical outcomes	222/811 (27%) patients developed AKI. The most common intensive care admission diagnosis in AKI cases was heart disease (38.6%). Hypoxic ischemia was the most frequent cause of AKI. Risk factors for AKI were multifactorial and were mainly associated with illness severity

*AKI, acute kidney injury; CKD, chronic kidney disease; DKA, diabetic ketoacidosis; DKD, diabetic kidney disease; T1D, type 1 diabetes; T2D, type 2 diabetes*.

### Markers of AKI in the Pediatric Population

The diagnosis of AKI, per the Kidney Disease Improving Global Outcomes (KDIGO) guidelines, is currently defined by factors including elevated serum creatinine concentration, decreased UO, and renal replacement therapy requirement (32, 33). UO is the oldest known indicator of AKI, yet it retains multiple theoretical advantages over biomarkers such as serum creatinine concentration due to a predefined cut-off value and rapid response to treatment. In contrast, serum creatinine concentration requires comparison to a baseline value which is often either unknown or estimated (34). However, the sole use of UO to diagnose AKI also has several constrains, notably the significant influence of hydration status and blood pressure, the dependence on hemodynamic stability, and the artificial effect of medications such as diuretics and vasopressors (34). Severe glycosuria from uncontrolled diabetes or use of SGLT2 inhibitors can also lead to prerenal or true AKI despite continued UO. Another issue includes the difficulty in accurately measuring UO, particularly in children without Foley catheters in place (34). UO is also not an infallible indicator of AKI as it has been shown that some individuals with severe AKI can still have a preserved UO (34). Serum creatinine concentration is also an imperfect indicator of AKI as it can be delayed and unreliable in the setting of concurrent infections, sepsis, malnutrition, and obesity, which can ultimately lead to an inaccurate diagnosis (34–36). Laboratory and clinical studies are currently targeting the identification of biomarkers that predict the development of early AKI. Some of the most promising AKI biomarkers include neutrophil gelatinase-associated lipocalin (NGAL), kidney injury molecule 1 (KIM-1), interleukin 18 (lL-18), liver-type fatty-acid-binding protein (L-FABP, also called FABP1), insulin-like-growth-factor-binding protein 7 (IGFBP7), and tissue inhibitor of metalloproteinase 2 (TIMP-2) (37, 38).

NGAL is a protease-resistant polypeptide of the lipocalin superfamily identified in human neutrophils and is expressed by the tubular epithelia of the kidneys in response to inflammation (39–41). NGAL is a promising marker for AKI because it is easily detected in urine or plasma as soon as 1 h following kidney damage and prior to changes in serum creatinine, and it correlates with the severity and duration of AKI (37). Additionally, urine NGAL has been extensively studied in pediatrics and has been shown to be an early and sensitive biomarker for both AKI and DKD (42).

KIM-1, a type-1 transmembrane protein expressed in the renal proximal tubular cells, is significantly upregulated following ischemia (43, 44). In a 2019 study by Assadi and Sharbaf urinary KIM-1 demonstrated the strongest performance for the early detection of AKI among critically ill children with circulatory collapse when compared to NGAL, IL-18, and sCr (45). However, while current evidence supports the use of KIM-1 as a promising new avenue for the detection of AKI, concentrations may be affected by a variety of additional factors including the type of assay, timing and clinical setting of the sample collection, and patient age (46). Similar to NGAL, KIM-1 has also been shown to be elevated in children with diabetes and higher concentrations may associated with the development of early DKD (47).

IL-18 is a cytokine in the IL-1 superfamily that is synthesized by monocytes, macrophages, and proximal tubular epithelial cells of the kidney. Urine IL-18 is currently a promising predictor of early AKI as concentrations increase rapidly after ischemic kidney injury, nearly 12 h before clinical AKI is diagnosed by other means (37). Yet, identification of additional biomarkers for AKI is still necessary as IL-18's accuracy in predicting AKI among children and adolescents is highest when combined with other biomarkers (37).

L-FABP is a cytoplasmic protein involved in intracellular lipid trafficking and endogenous cytoprotection against oxidative stress that is expressed in the proximal epithelial tubular cells of the kidneys (34, 48). Urine L-FABP has been shown to predict both AKI and adverse clinical outcomes in pediatrics, reflecting the negative impact of oxidative stress on the kidneys and the associated resultant proximal tubule cellular injury (38). However, urine L-FABP concentrations may be elevated by factors including obesity, insulin resistance, and high blood pressure, even without concurrent kidney injury (49).

IGFBP7 and TIMP-2 are cell-cycle arrest proteins expressed in higher concentrations from the renal tubular cells during cellular stress (50). Evaluations of the urinary excretion of TIMP-2 and IGFBP7 have been proven useful in the detection of AKI and the urinary concentration of the product of TIMP-2 and IGFBP7 [(TIMP-2) × (IGFBP7)] was the first urinary AKI biomarker approved by the United States Food and Drug Administration (51). Notably, this biomarker combination has also been validated in post-operative youth following cardiopulmonary bypass, demonstrating accuracy in predicting AKI 6 h after major surgery as well (38).

Other novel biomarkers are currently being investigated for the diagnosis and prognostic stratification of AKI in youth, including microRNAs, urinary low-molecular-weight proteins, and urinary tubular enzymes (37). The validation of these emerging biomarkers in large epidemiological studies is still necessary, but the biomarkers previously discussed hold significant clinical promise as supplements to our current tools for the identification of AKI in youth, namely serum creatinine concentration and UO.

### Pathophysiologic Mechanisms of AKI in Youth With Diabetes

**Figure 1:**
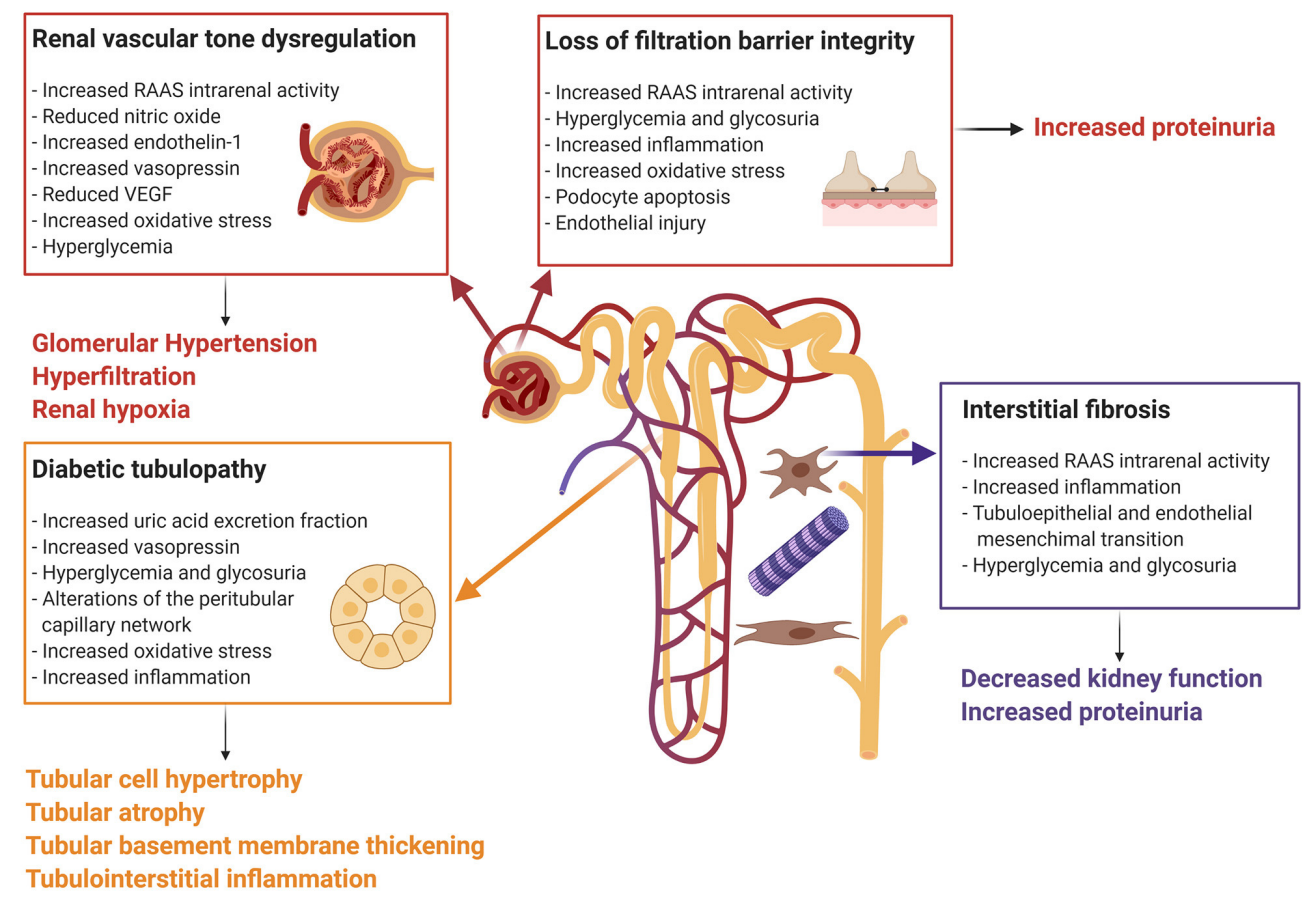
Mechanisms of diabetes-induced kidney injury. Every box represents one mechanism of diabetes-associated kidney injury with the respective causes. Connected to each box the clinical effect of the pathophysiologic pathway. The Figure was created with BioRender.com. RAAS, renin-angiotensin-aldosterone system; VEGF, vascular endothelial growth factor.

The pathophysiologic mechanisms leading to diabetes-induced kidney injury are complex and oftentimes multifactorial (Figure 1). Structural and functional changes in the endothelial cells of the vasculature and the epithelial cells of the kidney tubules have been postulated to promote the production of cytokines and chemokines, which induce inflammation, ischemic tubular epithelial and endothelial injury, and isolated proximal tubulopathy (19, 52, 53).

Risk factors for the development of AKI in children and adolescents with diabetes are numerous and include, but are not limited to, episodes of DKA as well as the presence of acute and chronic hyperglycemia. DKA is a severe risk factor for the development of AKI, particularly in youth with T1D, and is characterized by a combination of hyperglycemia, metabolic acidosis, and the production of ketone bodies (54, 55). DKA is currently the leading cause of hospitalization, morbidity, and mortality in youth with T1D (14, 56, 57) and in a study by Hursh et al. up to 64% of youth with T1D hospitalized for DKA developed AKI (14). This percentage increased to 85% if the individual required admission to the Intensive Care Unit (14). Severe hyperglycemia associated with DKA leads to osmotic diuresis, dehydration, and significant pre-renal AKI (54, 55).

Additionally, DKA has also been associated with early tubulopathy that may be caused by dysregulation of uric acid and vasopressin (58–62). Indeed, a recent study by Burckhardt et al. found that copeptin, a surrogate marker for vasopressin, was elevated in children with DKA (63). Elevated copeptin concentrations have also been associated with markers of tubular injury and an increased risk for the development of DKD (62, 64). We have previously demonstrated that adults with T1D and DKD had higher concentrations of copeptin compared to both healthy controls and adults with T1D without DKD (65). Furthermore, elevations in copeptin are more strongly correlated with intrarenal renin-angiotensin-aldosterone system (RAAS) activation in individuals with T1D compared to healthy controls (65). We have also demonstrated in a study of 169 adolescents with T1D that adolescents with T1D in the highest tertile of copeptin concentration had a significantly higher urinary albumin-to-creatinine ratio compared to those in the lowest tertile (66). Furthermore, several studies have shown that chronically elevated vasopressin can exacerbate kidney injury in animal models (67, 68).

Thus, we hypothesize that vasopressin dysregulation may increase the risk of AKI during an episode of DKA and may subsequently accentuate the risk for future DKD. Activation of the intrarenal RAAS has been shown to impact regulation of renal vascular tone and children and adolescents with T1D and T2D have demonstrated greater resistance indexes when compared to their healthy peers without diabetes (69–71). Additionally, nitric oxide (NO), endothelin-1 (ET-1), vasopressin, and vascular endothelial growth factor (VEGF) have also been shown to be impaired as regulators of kidney vascular tone in individuals with diabetes (58, 65, 72–74).

Dysregulation of kidney vascular endothelial tone secondary to poor glycemic control is thought to be primarily responsible for the development of glomerular hyperfiltration, a common early finding in young persons with diabetes (75, 76). Persistent glomerular hyperfiltration leads to the development of intraglomerular hypertension and subsequent glomerulosclerosis which results in a progressive impairment in kidney function and eventual DKD (75, 76). A dysregulation in baseline kidney vascular tone may accelerate the damage due to AKI, particularly in the setting of pre-renal AKI where the body relies on changes in kidney vascular resistance to maintain blood pressure (18, 19). Kidney hypoperfusion secondary to dehydration or shock also can be significantly worsened by the absence of an appropriate counterregulatory vascular response to maintain kidney blood flow (75, 76). Consequently, the incidence of AKI in the setting of sepsis has been found to be significantly higher in people with diabetes compared to people without diabetes (4, 77).

Hyperuricemia may also contribute to the development of diabetes-related acute and chronic kidney disease (60). High uric acid concentrations have been shown to induce both a crystal-mediated and crystal-independent nephropathy, with the additional development of tubulointerstitial fibrosis, proximal tubulopathy, and glomerular hypertension (61, 78). Elevations in serum uric acid concentrations have also been associated with the development of AKI and medications that lower serum uric acid concentrations have demonstrated a potential to reduce the incidence of AKI (79–82). However, it is important to note that elevated uric acid concentrations may be due to dehydration that may directly induce kidney injury. We have previously demonstrated that youth with T1D and hyperfiltration had a higher urinary fractional uric acid excretion compared to both healthy controls and peers with T1D who have normofiltration (81). Additionally, overweight and obese youth with T1D demonstrated a stronger negative correlation between serum uric acid concentrations and eGFR when compared to their normal weight peers with T1D (83). Thus, high serum uric acid concentrations exhibit a clear association with AKI. Additionally, not only do young persons with diabetes have higher baseline concentrations of serum uric acid, they also demonstrate a stronger association between elevations in uric acid concentration and impaired kidney function.

Another pathophysiologic pathway that has been shown to greatly contribute to the development of diabetes-induced AKI is the presence of persistent hyperglycemia, a hallmark of diabetes. The degree of persistent hyperglycemia has been shown to correlate with longer durations of stay in the intensive care unit as well as an increased risk for the development of AKI which can eventually lead to both CKD and ESKD (4, 12, 78). Notably, laboratory studies have demonstrated endothelial cell apoptosis, interstitial vascular rarefaction, mitochondrial dysfunction, proximal tubular cell inflammation, profibrogenic cytokine secretion, and podocyte apoptosis and autophagy in response to hyperglycemia (4).

Among the glomerular endothelial cells, hyperglycemia also induces cellular apoptosis via the Nuclear Factor-κB (NF-κB) and c-Jun NH2-terminal kinase (JNK) pathways (84, 85) and causes interstitial vascular rarefaction which leads to kidney hypoxic injury and can compromise the generation of necessary ATP (86). Overproduction of reactive oxygen species (ROS) has also been shown to induce endothelial cell damage and reduce the amount of nitric oxide available to modulate vascular tone, thereby contributing to dysregulation of sympathetic tone and sodium homeostasis in the kidneys (87).

Mitochondrial dysfunction has also been identified in the proximal tubules of individuals with diabetes during states of kidney hypoxia, further delaying future recovery from kidney injury (88). Furthermore, in diabetic rat models, the enzyme Myo-inositol oxygenase (MIOX) is upregulated in the setting of mitochondrial dysfunction, thereby inducing ROS production, cellular apoptosis, and eventually, DKD (89). MIOX is modulated by specificity protein-1 (Sp-1), a transcription factor which could serve as a potential site for the development of interventions targeting the progression of DKD as inhibition of Sp-1 has been shown to reduce ROS production (90).

Inflammatory cytokines in the proximal tubule have also been shown to play a significant role in the progression of AKI to diabetes-related CKD. Tissue necrosis factor (TNF)-α, interleukin (IL)-1, and IL-6 have been reported to be upregulated in the setting of diabetes-related CKD and are known to stimulate an inflammatory cascade that contributes to the progression of CKD (91–94). Hyperglycemia has also been shown to induce the expression of adhesion molecules and chemokines in proximal tubular cells that result in inflammation, fibrosis, and kidney injury (95, 96). Additionally, a maladaptive compensatory response for this hyperglycemia-induced inflammatory cascade in the proximal tubule leads to a profibrogenic cytokine secretion and subsequent fibrosis that plays a significant role in the transition of AKI to CKD (97). It has been demonstrated that proximal tubular cells cultured in hyperglycemic media secrete transforming growth factor beta (TGF-β)-dependent extracellular matrix (98, 99) and activation of this pathway, which plays a central role in mediating kidney fibrosis, has been shown to be significantly upregulated in both diabetic rat models and biopsies from humans with diabetes (100). Hyperglycemia has also induced podocyte apoptosis in murine models of diabetes and in adults with T2D, leading to kidney injury and future diabetes-related CKD. Podocyte apoptosis in the setting of hyperglycemia occurs through mechanisms that include, but are not limited to, increased ROS, RAAS activation, and upregulation of the mammalian target of rapamycin (mTOR) pathway (4, 101–105). However, unlike hyperglycemia-induced injury to the proximal tubule cells of the kidney, podocyte apoptosis in DKD is likely irreversible and leads to permanent damage (106).

### Risk of AKI in the Pediatric Population With Diabetes

Risk factors for AKI are numerous and include concurrent hospitalizations and comorbid conditions such as hypertension and diabetes. Diabetes is an important risk factor for AKI as it has been shown to independently associate with longer hospitalizations, increased severity of concomitant infections, and future development of CKD (5, 107–112). Indeed, there is a strong association between AKI, CKD, and ESKD (4, 6, 113–115), as it has been demonstrated that both single and repetitive episodes of AKI significantly increase the risk for developing CKD in youth and adults with diabetes (5–9). Additionally, the incidence of AKI is further compounded by factors including surgical interventions (116–120), aminoglycoside usage (121), and sepsis (4, 77), and this occurs to a greater degree in individuals with vs. without diabetes. Yet, while AKI and CKD remain strongly interconnected, as evidenced by a robust body of literature detailing the transition from AKI to CKD, the effects of AKI on CKD are less clear, thus resulting in a disproportionate focus on CKD vs. AKI in diabetes research (4, 13, 34).

## Therapeutic Strategies and Implications

To date, there is no universal effective therapy for AKI. As AKI is the result of different pathophysiologic mechanisms, the treatment consists of addressing the underlying causes (e.g., dehydration), correcting the hydro-electrolyte imbalances, withdrawing potential nephrotoxins, starting diuretics, and, when needed, renal replacement therapy (122). As previously mentioned, children with diabetes and DKD have a higher risk of developing AKI. In this paragraph we will discuss how DKD therapeutic strategies may impact the risk of AKI in youth with diabetes.

RAAS inhibitors, in addition to strategies that target euglycemia and minimize cardiovascular risk factors, remain the cornerstone of our existing treatment methods for DKD and has shown to attenuate proteinuria (123–125). In the SEARCH for Diabetes in Youth (SEARCH) study, youth with T2D were found to have significantly higher urinary albumin-to-creatinine ratios than youth with T1D (126), and this difference remained significant after multivariable adjustment for obesity, hypertension, and dyslipidemia, all known risk factors for DKD that are more prevalent in youth with T2D vs. T1D (126). Consequently, due to known differences in risk for the development of DKD, treatment of youth with T2D with RAAS inhibitors may be more frequently indicated than treatment of youth with T1D. However, RAAS inhibitors should be withdraw during AKI due to their effects on intrarenal hemodynamic function as well as potential direct nephrotoxic effect, which could magnify the acute kidney insult (123–125).

In addition to glucose-lowering effects, many anti-hyperglycemic agents available today also showcase significant nephroprotective properties in DKD. To date, anti-hyperglycemic agents that have demonstrated nephroprotective properties include metformin and thiazolidinediones, as well as newer classes of glycemic-lowering agents including dipeptidyl peptidase-4 (DPP-4) inhibitors, glucagon-like peptide-1 (GLP-1) receptor agonists, and sodium/glucose cotransporter 2 (SGLT2) inhibitors (127–129). Insulin, metformin and GLP-1 receptor agonists are the only medications approved for diabetes treatment in children in the US. Despite being widely used in the of the management of patients with diabetes-related CKD, metformin has been shown to accumulate in the setting of an impaired eGFR (e.g., 30–60 mL/min/1.73 m^2^), thereby causing toxicity that can lead to impairments in mitochondrial function and possible non-hypoxic type B lactic acidosis (130). Thiazolidinediones, such as pioglitazone, are insulin sensitizing agents that target Peroxisome Proliferators Activated Receptors (PPARs) to regulate gene expression and thereby decrease hepatic gluconeogenesis, increase adiponectin concentrations, and increase insulin-dependent glucose uptake in muscle and fat tissues (131). Studies have previously demonstrated a possible role for PPARs in the protection against AKI (132–134) and a large meta-analysis reported an association between treatment with thiazolidinediones and a significant decrease in urinary albumin excretion (129). Yet, no randomized controlled trials have been completed to thoroughly explore the acute and chronic kidney protective effects of thiazolidinediones (135). Next, some studies have also suggested beneficial nephroprotective effects for both DPP-4 inhibitors and GLP-1 receptor agonists (127, 136). However, a recent Cochrane review was less definitive and concluded that the known effects of DPP-4 inhibitors and GLP-1 receptor agonists on eGFR are uncertain (136). Additionally, SGLT2 inhibitors have been shown to be beneficial in the prevention of AKI in two different randomized, placebo-controlled trials: Empagliflozin Cardiovascular Outcome Event Trial in Type 2 Diabetes Mellitus Patients (EMPA-REG OUTCOME) and Dapagliflozin Effect on Cardiovascular Events-Thrombolysis in Myocardial Infarction 58 (DECLARE-TIMI 58) (137, 138). However, similarly to DPP-4 inhibitors and GLP-1 receptor agonists, a recent Cochrane review found that SGLT2 inhibitors had little to no effect on the risk for AKI (136).

Several ongoing or recently terminated trials have also analyzed the effects of other classes of drugs, particularly those targeting the kidney vasculature and the complex homeostatic mechanisms of vasoconstriction and vasodilation, in the prevention and treatment of DKD (74). Several compounds have shown promise with possible nephroprotective effects against both AKI and CKD; however, many of the human trials are still ongoing and most exclude pediatric participants. In animal models, endothelin receptor antagonists, PPAR agonists, phosphodiesterase inhibitors, and novel mineralocorticoid receptor blockers have demonstrated possible nephroprotective effects against AKI and may represent future therapeutic strategies for the prevention and treatment of AKI and CKD (123, 139–142). In particular, finerenone, a non-steroidal selective mineralocorticoid receptor antagonist, has recently been shown to reduce the risk of CKD progression in adults with T2D in the Efficacy and Safety of Finerenone in Subjects with Type 2 Diabetes Mellitus and Diabetic Kidney Disease (FIDELIO-DKD) study (143). Notably, finerenone was also able to prevent the transition from AKI to CKD in animal models and could represent an early therapeutic intervention for individuals demonstrating AKI to prevent long term complications (144).

In summary, in addition to known anti-hyperglycemic effects, many glucose-lowering agents have also demonstrated evidence of nephroprotection against both diabetes-associated AKI and CKD, two comorbidities that significantly increase the risk of morbidity and mortality in youth with diabetes. Additionally, newer classes of drugs that target intrarenal hemodynamic dysfunction in children and adolescents with diabetes are a promising new avenue for the treatment of both AKI and CKD. Consequently, well-designed pediatric trials are warranted to address the steadily increasing burden of AKI in children and adolescents with diabetes and subsequently prevent the development and progression of chronic DKD.

## Covid-19 Impact

Epidemiologic studies have demonstrated evidence of AKI in more than 35% of adults who are hospitalized for the novel coronavirus disease 2019 (COVID-19) (145, 146). The prevalence of diabetes in patients with COVID-19 varies from 5 to 58% (147), yet patients with diabetes are far more likely to develop severe COVID-19 and consequent AKI than their counterparts without diabetes, with a prevalence of critical disease varying from 14 to 32% in adults with diabetes and COVID-19 (147). Acute proximal tubular damage, collapsing glomerulopathy, and podocyte injury have been found to be pathologic hallmarks of kidney injury in adults with confirmed COVID-19 (148–150).

In contrast, AKI in youth with COVID-19 has mostly been described as a consequence of dehydration secondary to gastrointestinal side effects (151, 152). One possible explanation that has been proposed is the presence of immature angiotensin converting enzyme 2 (ACE2) receptors in children and adolescents (151). ACE2 receptors are expressed in both the proximal tubular cells and podocytes and thus may be a site of cytokine-mediated effects as a result of a heightened immune system response in the setting of an active COVID-19 infection (149, 153). However, COVID-19-induced effects on the ACE2 receptor may not fully describe the AKI seen in children and adolescents with COVID-19 as other factors including the presence of a cytokine storm and/or a pro-coagulative state have also been shown to be independently associated with AKI in the setting of COVID-19 (145, 151). This finding has been corroborated by evidence of a close temporal relationship between the development of AKI and impending respiratory failure in patients with COVID-19 (145).

Thus, while AKI remains a somewhat rare complication of COVID-19 infection in pediatrics, it is notable that a concurrent diagnosis of diabetes increases the risk of associated kidney injury. Methods to assist with rapid diagnosis and treatment of AKI in youth, particularly in those with diabetes, could prevent further AKI progression and help mitigate the risk of future CKD development.

## Author Contributions

FP, TR, and PB contributed to the conception and design of the review paper. All authors contributed to the manuscript revision, read, and approved the submitted version.

## Conflict of Interest

PB has acted as a consultant for AstraZeneca, Bayer, Bristol-Myers Squibb, Boehringer Ingelheim, Sanofi, Novo Nordisk, Lilly USA, and Horizon Pharma. PB serves on the advisory board of AstraZeneca, NovoNordisk, XORTX, and Boehringer Ingelheim. KT receives salary and research support from the NIDDK (5T32DK007135-43), ISPAD-JDRF Research Fellowship, Center for Women's Health Research at the University of Colorado, and the Department of Pediatrics, Section of Endocrinology at the University of Colorado School of Medicine. RJ has consulted for Horizon Pharma and also has equity with XORTX Therapeutics and with Colorado Research Partners, LLC. The remaining authors declare that the research was conducted in the absence of any commercial or financial relationships that could be construed as a potential conflict of interest.
